# Supplementary material for: Multi-trait discovery and fine-mapping of lipid loci in 125,000 individuals of African ancestry
Source: Nat Commun. 2023 Sep 5;14:5403. doi: 10.1038/s41467-023-41271-0 (PMC10480211; doi:10.1038/s41467-023-41271-0)
Supplement: Supplementary file 1 — Supplementary Information [file 41467_2023_41271_MOESM1_ESM.pdf]

Supplementary Information

Multi-trait discovery and fine-mapping of lipid loci in 125,000 individuals of African ancestry

Abram Bunya Kamiza[1,2,3,*], Sounkou M, Touré [1,4,*], Feng Zhou[5,*], Opeyemi Soremekun[1], Cheickna Cissé [4,7], Mamadou Wélé [2,7], Aboubacrine M. Touré [7], Oyekanmi Nashiru [6], Manuel Corpas[8], Moffat Nyirenda[9], Mia Crampin[2], Jeffrey Shaffer[10], Seydou Doumbia [4,11], Eleftheria Zeggini[12,13], Andrew P Morris[14], Jennifer L Asimit[5], Tinashe Chikowore [3,15], Segun Fatumo [1,6,12,16]

[1]The African Computational Genomic (TACG) Research Group, MRC/UVRI and LSHTM, Uganda
[2]Malawi Epidemiology and Intervention Research Unit, Lilongwe, Malawi
[3]Sydney Brenner Institute for Molecular Bioscience, Faculty of Health Sciences, University of the Witwatersrand, Johannesburg, South Africa
[4]African Center of Excellence in Bioinformatics, University of Sciences, Techniques and Technologies of Bamako, Bamako, Mali
[5]MRC Biostatistics Unit, University of Cambridge, UK
[6]H3Africa Bioinformatics Network (H3ABioNet) Node, Center for Genomics Research and Innovation, NABDA/FMST, Abuja
[7]Faculty of Sciences and Techniques, University of Sciences, Techniques and Technologies of Bamako, Bamako, Mali
[8]University of Westminister, London
[9]Medical Research Council/Uganda Virus Research Institute and London School of Hygiene & Tropical Medicine Uganda Research Unit
[10]Department of Biostatistics and Data Science, School of Public Health and Tropical Medicine, Tulane University, New Orleans, Louisiana, United States of America
[11]Faculty of Medicine and Odonto-stomatology, University of Sciences, Techniques and Technologies of Bamako, Bamako, Mali
[12]Institute of Translational Genomics, Helmholtz Zentrum München - German Research Center for Environmental Health, Neuherberg, Germany
[13]TUM School of Medicine, Translational Genomics, Technical University of Munich and Klinikum Rechts der Isar, Munich, Germany
[14]Centre for Genetics and Genomics Versus Arthritis, Centre for Musculoskeletal Research, University of Manchester, Manchester, UK
[15]MRC/Wits Developmental Pathways for Health Research Unit, Department of Pediatrics, Faculty of Health Sciences, University of the Witwatersrand, Johannesburg, South Africa
[16]Department of Non-Communicable Disease Epidemiology, London School of Hygiene and Tropical Medicine, London, United Kingdom

* These authors contributed equally.

Supplementary Fig.1 Forest plot showing a comparison of some genetic variants. APCDR: African Partnership for Chronic Disease Research consortium in Africa (N=~14,000). AWI-Gen; Africa Wits-INDEPTH Partnership for Genomic Research (N=~11,000). GLGC; Global lipid genetic consortium of African ancestry (N=~99,000). P: Two-tailed p-value, not adjusted for multiple comparisons, developed using R. The error bars show odds ratio ± 95% confidence intervals.

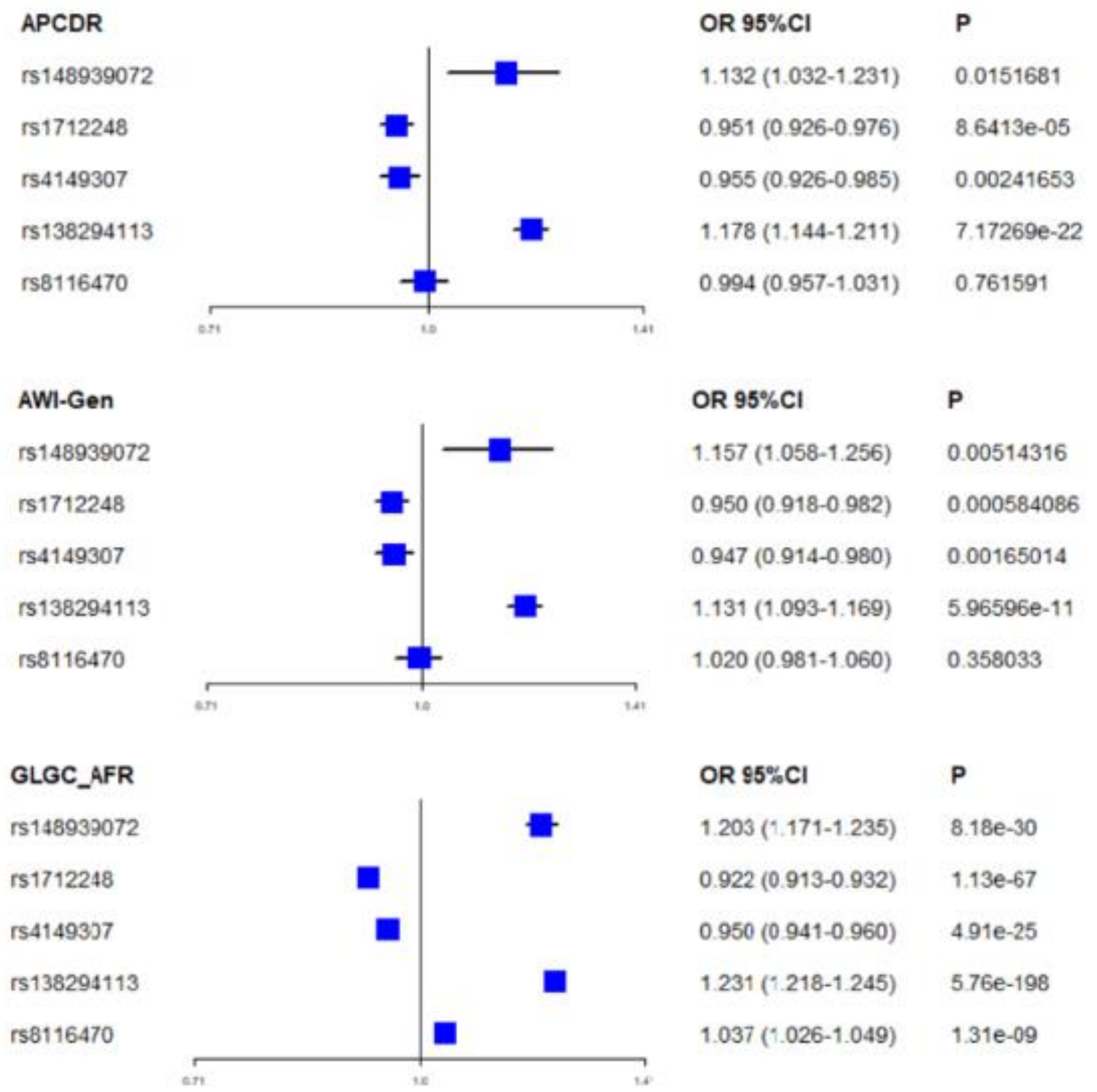

Supplementary Fig.2 Locus zoom plot for top novel loci associated with lipid traits in individuals of African ancestry from the MTAG analysis (N=~125,000). (**a**) Locuszoom plot showing associations around the *TMEM64* region. **(b)** Locuszoom plot showing associations around *ZNF782* region. (**c**) Locuszoom plot showing associations around the intergenic region between *MSANTD3* and *TMEFF1*. (**d**) Locuszoom plot showing associations around the *LOC100507346* region.

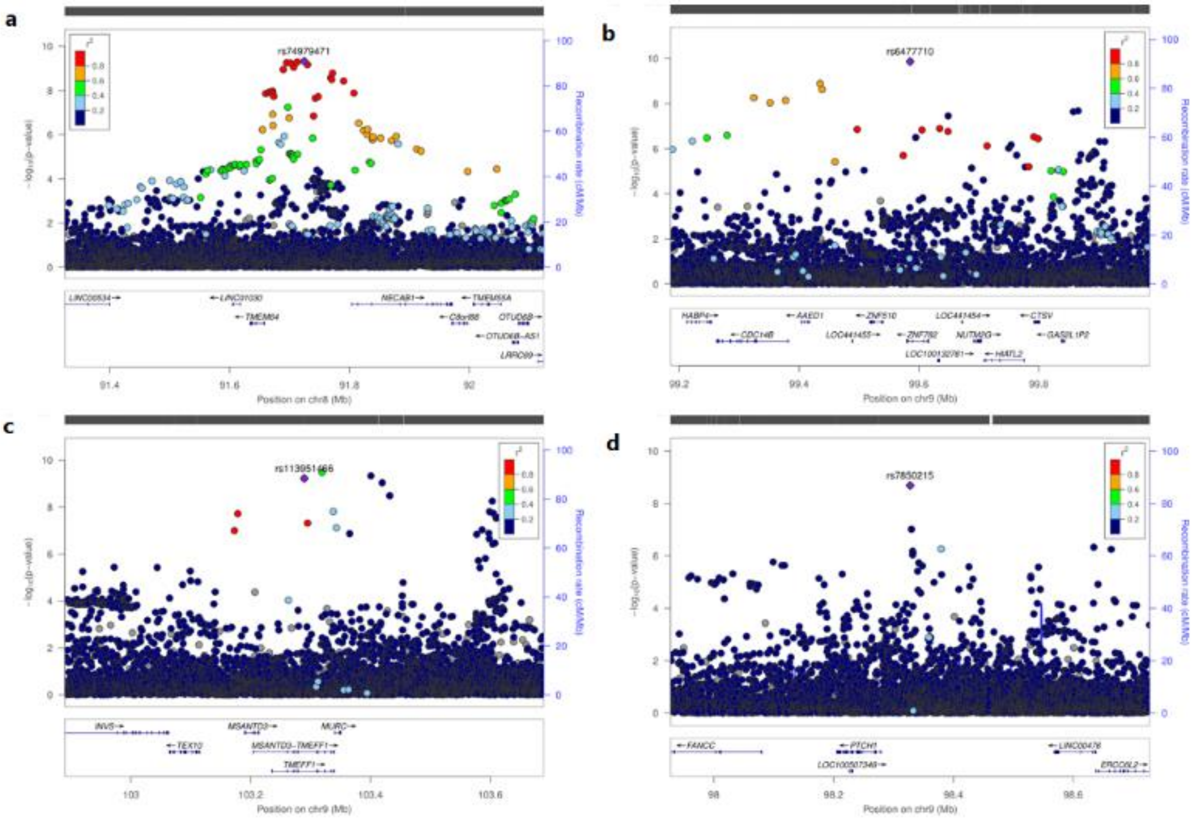

Supplementary Fig.3 Venn diagram showing the number of independent genetic loci associated with lipid traits identified by meta-analysis and MTAG approach in individuals of African ancestry. The blue colour is the number of independent genetic loci for meta-analysis GWAS and yellow color is the number of independent genetic loci for MTAG analysis. The overlapping area between blue and yellow are number of loci shared between meta-analysis GWAS and MTAG. HDL; high-density lipoprotein, TG: triglycerides, TC: total cholesterol.

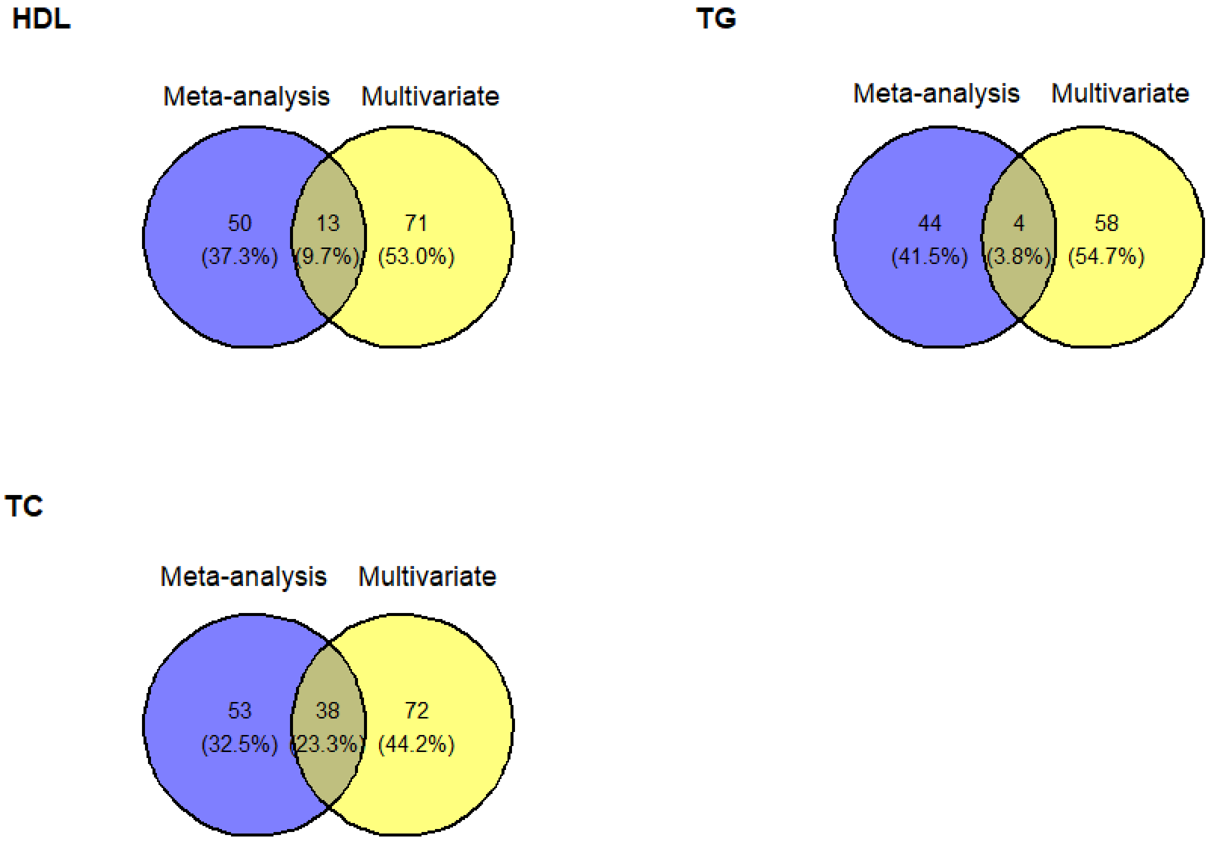

Supplementary Fig.4 Regional association plots of 16:56889590-57089590 for LDL and TG, integrated with fine-mapping MPP. (**a**) rs247616 with marginal posterior probability of causality (MPP) =0.501 for single trait LDL. (**b**) rs183130 with MPP= 0.999 of causality with mult-itrait LDL. (**c**) rs4783961 with MPP=0.332 of causality for single trait TG. (**d**) rs183130 with MPP=0.999 of causality for multi-trait TG. We display p-values by location height (y-axis) and MPP by diameter of the points. Variants that are the same colour may be thought of as interchangeable and are said to belong to the same SNP group; variants in different groups have r^2>0.6 and rarely appear in a model together. This figure was generated using the web tool flashfm-ivs (http://shiny.mrc-bsu.cam.ac.uk/apps/flashfm-ivis/)

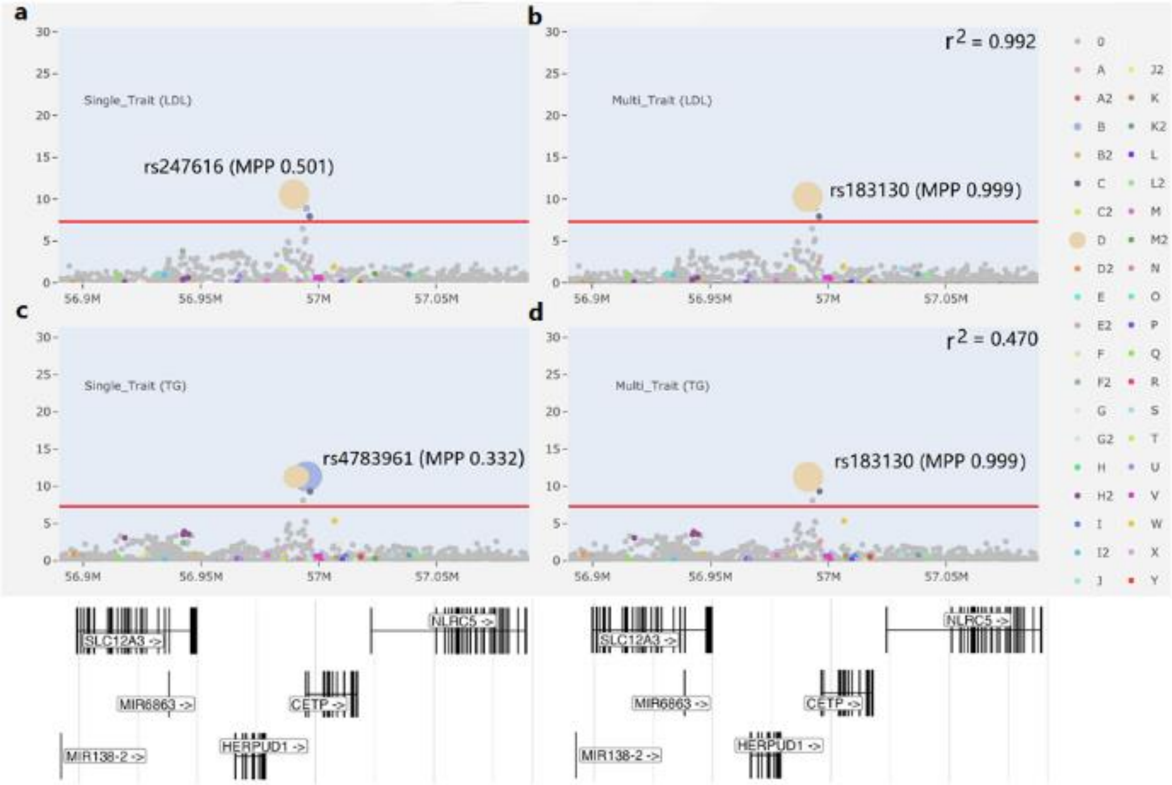

Supplementary Fig.5 Regional association plots of 21:46753876-46975775 for TC, TG, and HDL, integrated with fine-mapping. (**a**) rs77974343 with marginal posterior probability of causality (MPP) =0.275 for single trait TC. (**b**) rs116386571 with MPP= 0.989 of causality with mult-itrait TC (**c**) rs116386571 with MPP=1 of causality for single trait TG. (**d**) rs116386571 with MPP=1 of causality for multi-trait TG. (**e**) rs116386571 with MPP=0.863 of causality for single trait HDL.(**f**) rs116386571 with MPP=0.999 of causality for multi-trait HDL. We display p-values by location height (y-axis) and MPP by diameter of the points. We display p-values by location height (y-axis) and MPP by diameter of the points. Variants that are the same colour may be thought of as interchangeable and are said to belong to the same SNP group; variants in different groups have r^2>0.6 and rarely appear in a model together. This figure was generated using the web tool flashfm-ivs (http://shiny.mrc-bsu.cam.ac.uk/apps/flashfm-ivis/)

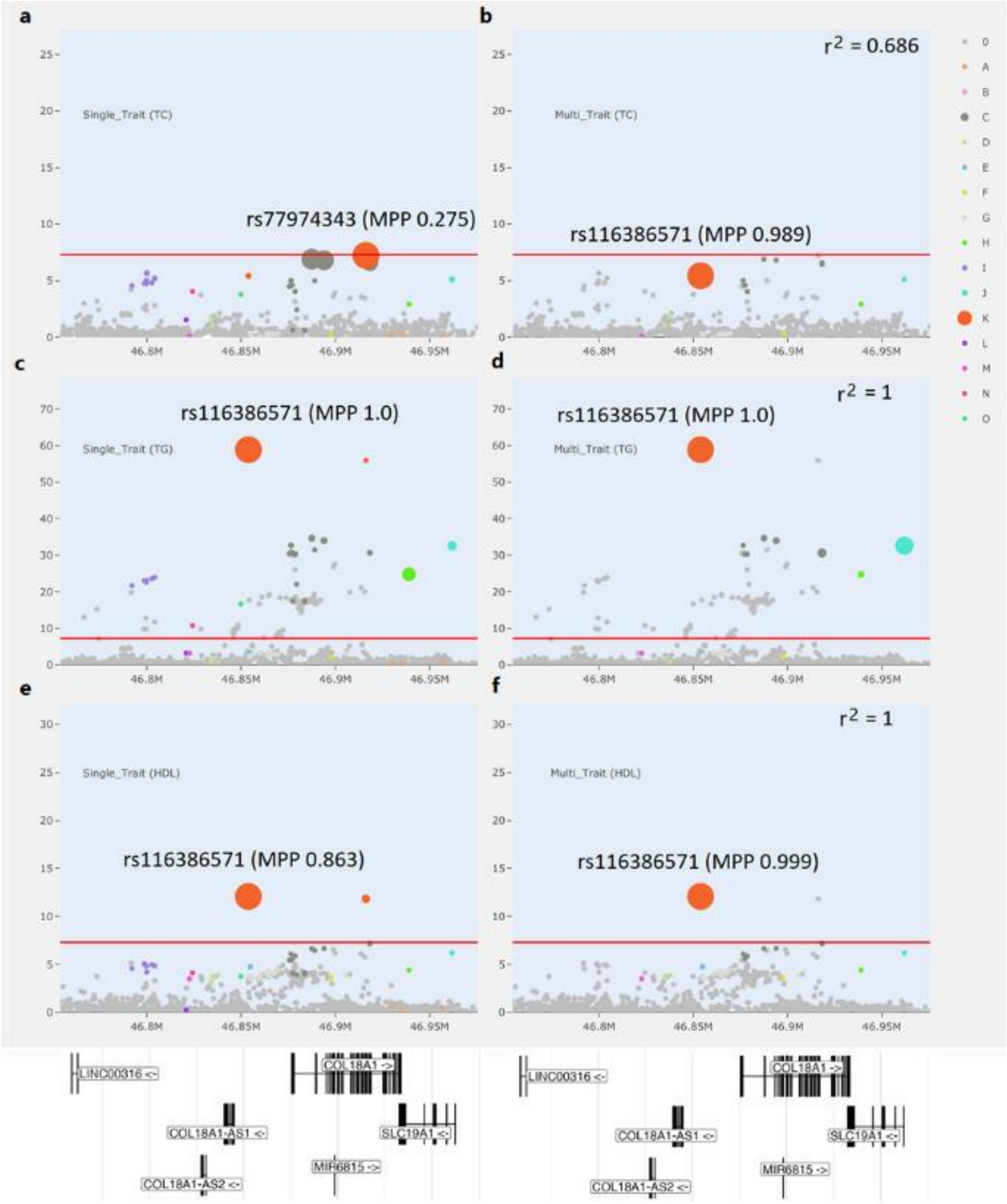
a
b
c
d
e
f
Single_Trait (TC)
Multi_Trait (TC)
Single_Trait (TG)
Multi_Trait (TG)
Single_Trait (HDL)
Multi_Trait (HDL)
rs77974343 (MPP 0.275)
rs116386571 (MPP 0.989)
r2 = 0.686
rs116386571 (MPP 1.0)
rs116386571 (MPP 1.0)
r2 = 1
rs116386571 (MPP 0.863)
rs116386571 (MPP 0.999)
r2 = 1
46.8M
46.85M
46.9M
46.95M
LINC00316 <-
COL18A1 ->
COL18A1-AS1 <-
SLC19A1 <-
MIR6815 ->
COL18A1-AS2 <-
