# Supplementary material for: Multi-trait discovery and fine-mapping of lipid loci in 125,000 individuals of African ancestry
Source: Nat Commun. 2023 Sep 5;14:5403. doi: 10.1038/s41467-023-41271-0 (PMC10480211; doi:10.1038/s41467-023-41271-0)
Supplement: Supplementary file 3 — Description of Additional Supplementary Files [file 41467_2023_41271_MOESM3_ESM.pdf]

Description of Additional Supplementary Files

File Name: Supplementary Data 1
Description: Independent genetic loci associated with lipid trait in individuals of African ancestry (N=~125,000). P-values are two-tailed calculated using GWAMA and not adjusted for multiple comparisons.

File Name: Supplementary Data 2
Description: Independent genetic loci associated with HDL, TG and TC in individuals of African ancestry (N=~125,000). P-values are two-tailed calculated using MTAG and not adjusted for multiple comparisons.

File Name: Supplementary Data 3
Description: Comparison of MTAG novel loci among individuals of African ancestry, GLGC European and East Asian ancestry.

File Name: Supplementary Data 4
Description: Fine mapping between multi-trait fine-mapping and single-trait fine-mapping

File Name: Supplementary Data 5
Description: Details of variants prioritised by JAM or flashfm for the lipids traits, including nearest genes, functional annotations, and prioritisation status in the GLGC analyses

File Name: Supplementary Data 6
Description: Colocalization of novel meta-analysis of genome-wide association studies with expression quantitative trait loci GTEx v8

File Name: Supplementary Data 7
Description: Colocalization of novel multi-trait analysis of genome-wide association studies analysis with expression quantitative trait loci GTEx v8
