# Supplementary material for: Multi-trait discovery and fine-mapping of lipid loci in 125,000 individuals of African ancestry
Source: Nat Commun. 2023 Sep 5;14:5403. doi: 10.1038/s41467-023-41271-0 (PMC10480211; doi:10.1038/s41467-023-41271-0)
Supplement: Supplementary file 11 — Reporting Summary [file 41467_2023_41271_MOESM11_ESM.pdf]

nature portfolio

Corresponding author(s): Segun Fatumo

Last updated by author(s): 07-August-2023

Reporting Summary

Nature Portfolio wishes to improve the reproducibility of the work that we publish. This form provides structure for consistency and transparency in reporting. For further information on Nature Portfolio policies, see our Editorial Policies and the Editorial Policy Checklist.

Statistics

For all statistical analyses, confirm that the following items are present in the figure legend, table legend, main text, or Methods section.

n/a	Confirmed	
☐	☒	The exact sample size (*n*) for each experimental group/condition, given as a discrete number and unit of measurement
☒	☐	A statement on whether measurements were taken from distinct samples or whether the same sample was measured repeatedly
☐	☒	The statistical test(s) used AND whether they are one- or two-sided<br>*Only common tests should be described solely by name; describe more complex techniques in the Methods section.*
☐	☒	A description of all covariates tested
☐	☒	A description of any assumptions or corrections, such as tests of normality and adjustment for multiple comparisons
☐	☒	A full description of the statistical parameters including central tendency (e.g. means) or other basic estimates (e.g. regression coefficient) AND variation (e.g. standard deviation) or associated estimates of uncertainty (e.g. confidence intervals)
☐	☒	For null hypothesis testing, the test statistic (e.g. *F*, *t*, *r*) with confidence intervals, effect sizes, degrees of freedom and *P* value noted<br>*Give P values as exact values whenever suitable.*
☐	☒	For Bayesian analysis, information on the choice of priors and Markov chain Monte Carlo settings
☐	☒	For hierarchical and complex designs, identification of the appropriate level for tests and full reporting of outcomes
☐	☒	Estimates of effect sizes (e.g. Cohen's *d*, Pearson's *r*), indicating how they were calculated

Our web collection on statistics for biologists contains articles on many of the points above.

Software and code

Policy information about availability of computer code

Data collection	No software was used for data collection
Data analysis	PLINK 1.9, Genome-wide association meta-analysis (GWAMA),Multi-trait Analysis of GWAS (MTAG), flashfm-ivs (http://shiny.mrc-bsu.cam.ac.uk/apps/flashfm-ivis/), JAM, LocusZoom, FUMA (https://fuma.ctglab.nl/)

For manuscripts utilizing custom algorithms or software that are central to the research but not yet described in published literature, software must be made available to editors and reviewers. We strongly encourage code deposition in a community repository (e.g. GitHub). See the Nature Portfolio guidelines for submitting code & software for further information.

Data

Policy information about availability of data

All manuscripts must include a data availability statement. This statement should provide the following information, where applicable:

- Accession codes, unique identifiers, or web links for publicly available datasets
- A description of any restrictions on data availability
- For clinical datasets or third party data, please ensure that the statement adheres to our policy

The genome-wide association summary statistics data used in this study are publicly available at https://www.ebi.ac.uk/gwas/downloads/summary-statistics. The summary statistic data generated have been deposited in the GWAS Catalog database under accession codes: GCST90278110 for HDL, GCST90278111 for LDL,

GCST90278112 for TG and GCST90278113 for TC. The processed data generated in this study are provided in the Supplementary Information and Supplementary Data.

Research involving human participants, their data, or biological material

Policy information about studies with human participants or human data. See also policy information about sex, gender (identity/presentation), and sexual orientation and race, ethnicity and racism.

Reporting on sex and gender	All GWAS summary data utilized in this meta-analysis contained information on self-reported sex. Nonetheless, we did not conduct a sex-stratified analysis as we did not have access to individual genotyped data.
Reporting on race, ethnicity, or other socially relevant groupings	The study is reporting findings in individuals of African ancestry in the US, UK, and continental Africa.
Population characteristics	The majority of the participants included in the study were between the ages of 40-60 and comprised both males and females. Along with self-reported age and sex, the study also collected the identity of the participants, as well as blood samples for lipid trait measurement and genotyping. The characteristics of the participants, including covariates, are included in the supplementary data.
Recruitment	We used genome wide association summary statistics data from participants were recruited from APCDR, GLGC_AFR and AWI-Gen.
Ethics oversight	The study was approved by the Uganda Virus Research Institute, Research and Ethics Committee (GC/127/944)

Note that full information on the approval of the study protocol must also be provided in the manuscript.

Field-specific reporting

Please select the one below that is the best fit for your research. If you are not sure, read the appropriate sections before making your selection.

☒ Life sciences ☐ Behavioural & social sciences ☐ Ecological, evolutionary & environmental sciences

For a reference copy of the document with all sections, see nature.com/documents/nr-reporting-summary-flat.pdf

Life sciences study design

All studies must disclose on these points even when the disclosure is negative.

Sample size	Summary data of 125,000 individuals of African ancestry were analysed in this study. Of these individuals ~14,000 were from the African Partnership for Chronic Disease Research (APCDR) consortium in Africa, ~99,000 were from the GLGC and ~ 11,000 were from the Africa Wits-INDEPTH partnership for Genomic Studies (AWI-Gen). No specific calculations were performed to determine the sample size. Nevertheless our sample size had enough power(85%) of detecting an association between a genetic factor and lipid trait.
Data exclusions	We excluded individuals and single nucleotide polymorphisms failing the stringent quality control procedures.
Replication	The replication was carried out by observing whether the same signal identified in our study was observed in other GWAS cohorts, such as the GLGC and AWI-GEN. We observed replication of many of the signals detected in our study from the aforementioned cohorts.
Randomization	We did not allocate any samples to groups, hence randomization was not relevant in this study,
Blinding	The study does not involve treatment, hence blinding was not relevant.

Reporting for specific materials, systems and methods

We require information from authors about some types of materials, experimental systems and methods used in many studies. Here, indicate whether each material, system or method listed is relevant to your study. If you are not sure if a list item applies to your research, read the appropriate section before selecting a response.

Materials & experimental systems

n/a	Involved in the study
☒	☐ Antibodies
☒	☐ Eukaryotic cell lines
☒	☐ Palaeontology and archaeology
☒	☐ Animals and other organisms
☒	☐ Clinical data
☒	☐ Dual use research of concern
☒	☐ Plants

Methods

n/a	Involved in the study
☒	☐ ChIP-seq
☒	☐ Flow cytometry
☒	☐ MRI-based neuroimaging
